# Preferences for genetic interventions for SCA and Huntington’s disease: results of a discrete choice experiment among patients

**DOI:** 10.1186/s13023-024-03408-2

**Published:** 2024-10-28

**Authors:** Nienke J.H. van Os, Mayke Oosterloo, Janneke P.C. Grutters, Brigitte A.B. Essers, Bart P.C. van de Warrenburg

**Affiliations:** 1 Department of Neurology, Radboud University Medical Center, Nijmegen, the Netherlands; 2 Department of Neurology, Maastricht University Medical Centre+, Maastricht, the Netherlands; 3 School of Mental Health and Neuroscience, Faculty of Health, Medicine and Life Sciences, Maastricht University, Maastricht, The Netherlands; 4 Science Department IQ Health, Radboud University Medical Center, Nijmegen, the Netherlands; 5 Department of Clinical Epidemiology and Medical Technology Assessment, Maastricht University Medical Centre+, Maastricht, the Netherlands

## Abstract

**Background:**

Although genetic interventions are on the horizon for some polyglutamine expansion diseases, such as subtypes of spinocerebellar ataxia (SCA) and Huntington’s disease (HD), the patients’ preferences regarding these new therapies are unclear. This study aims to get insight into what extent different characteristics of genetic interventions affect the preferences of patients with SCA and HD with regard to these interventions.

**Methods:**

Manifest and premanifest patients with SCA or HD were recruited online by platforms of patient associations. The respondents conducted a questionnaire that included a discrete choice experiment (DCE). The experimental design included 24 choice sets, but these were divided into three blocks of eight to reduce the number of tasks per respondent. Each choice set included two alternative treatments and consisted of four attributes (mode and frequency of administration, chance of a beneficial effect, risks, and follow-up), each with three or four different levels. The forced choice-elicitation format was used. Data were analyzed by using a multinominal logistic regression model.

**Results:**

Responses of 216 participants were collected. The mode and frequency of administration of a genetic intervention, as well as the chance of a beneficial effect both influence the choice for a genetic intervention. Respondents less prefer repeated lumbar punctures compared to a single operation. As expected, a higher beneficial effect of treatment was preferred. Risks and follow-up did not influence the choice for a genetic intervention.

**Conclusions:**

The results can be used for the design and implementation of future genetic interventional trials as well as of patient-centered care pathways for rare movement disorders such as SCA and HD.

**Supplementary Information:**

The online version contains supplementary material available at 10.1186/s13023-024-03408-2.

## Introduction

Spinocerebellar ataxia (SCA) types 1, 2, 3, 6, 7, and 17, and Huntington’s disease (HD) are genetic neurodegenerative diseases caused by trinucleotide CAG repeat expansions in different disease-specific genes [1, 2]. Expansions of the polyglutamine (polyQ) tract in the disease-causing protein lead to a toxic gain-of-function. Although the underlying molecular mechanisms between SCA and HD overlap, the classic phenotype of both diseases differs. Patients with SCA develop a cerebellar syndrome, in some forms accompanied by non-ataxia features such as extrapyramidal movement disorders, polyneuropathy, ocular problems, spasticity, and cognitive decline [3]. Patients with HD develop chorea, and psychiatric or behavioral problems with dementia [4].

To date SCA and HD are both progressive and incurable, but new treatments for these diseases are being developed and clinically tested [5–7]. Genetic interventions are promising, as they are designed to reduce levels of the disease-causing protein by silencing transcriptional or modulating translational processes through micro-RNAs or antisense-oligonucleotides (AON) [8]. As these agents cannot cross the blood-brain barrier, they need to be administered through repeated intrathecal or single intracerebral injections. At this moment, these therapies are being studied in different clinical phase 1 to 3 trials [6, 9–12]. The long-term benefits and possible risks for individual patients are not yet clear [13].

Knowledge of the patients’ perspective on these therapies is of major relevance in patient-centered healthcare. In general, patient engagement can make important contributions to the customization of trial designs and clinical care pathways. This is particularly important for rare genetic diseases such as SCA and HD, as unmet needs and relevant endpoints of therapies are often not known. Patients are very engaged and can help prioritizing certain therapeutic developments and reflect on risks versus benefits [14]. For HD, it is known that complex trade-offs between pros and cons occur in patients and their family members, while considering new treatments [15]. By involving patients in this process, patients are more satisfied and self-manageable [16], and compliance and success rates of new therapies can improve [17]. In this study, we aimed to identify to what extent different characteristics of genetic interventions affect the preferences of patients with SCA and HD, and what the relative importance of these characteristics is. We used a discrete choice experiment (DCE), a method to quantify the strength of patients’ preferences regarding different aspects of a treatment. In a DCE, participants are requested to repeatedly choose between two hypothetical treatments, both with different characteristics [18, 19].

## Methods

### Discrete choice experiment

A discrete choice experiment (DCE) was used, a technique that describes an intervention or therapy by its attributes like effectiveness, side effects or costs, and their levels. The combinations of different attributes and levels are used to characterize a number of hypothetical treatment choice sets. For every choice set, a participant is asked to choose the option they prefer [18, 19]. The aim is to establish which characteristics of genetic interventions influence choice behavior and which characteristics are preferred. For this study, the ISPOR guideline for conjoint analysis was used [20].

### Identification and selection of the attributes

For the identification of the attributes, the following steps were taken. First, literature was reviewed for potentially relevant attributes related to genetic interventions for SCA and HD. A search in the PubMed database was performed in December 2021 and combined search terms for ‘genetic therapy’, with terms for ‘patient’, ‘perspectives’, and terms for SCA and HD. Since the search led to only two relevant results, the search was extended by also including studies of other neurodegenerative disorders. In total, four papers were eligible and data regarding different characteristics of genetic interventions were extracted in an Excel spreadsheet [21–24].

Second, a list of possible relevant attributes was made by the first author, based on the results of the literature review. For the first version of the list, all characteristics of genetic interventions described in the four papers were included. This list of possible relevant attributes was discussed within the research team and consensus was reached about the attributes that were eligible for inclusion in the final list of topics (such as treatment goals and advantages, risks of procedures, treatment procedures, timing of treatment, and trial participation) for the semi-structured interviews with patients. Inclusion was based on the degree of occurrence in the papers and clinical and contextual relevance. Almost all characteristics were included in the list of topics for the interviews. The aim of the semi-structured interviews was to identify the most relevant attributes as seen by patients.

Ten patients (five with HD and five with SCA; seven manifest, one early manifest and two premanifest) were recruited to participate in semi-structured interviews. All patients gave written informed consent. The interviews were guided by the list of attributes and were conducted by phone or video conference in December 2021 or January 2022. Since saturation was achieved at the end of the 10 interviews, no further interviews with other patients were planned. Detailed results of these interviews are published separately [25]. In general, patients were asked for reasons to undergo genetic interventions and for reasons not to undergo genetic interventions. Furthermore, their opinions on logistic and social factors such as type of intervention, time investment, location, expertise, timing, and opinion of others, were explored.

The main results of the interviews were used to establish a list of attributes that seemed to be most relevant in the decision making process of patients. This list of attributes was discussed within the research team until consensus was reached about the final list. The final attributes needed to be clear and needed to have levels that are compatible with the DCE design. The final list included four attributes: [1] mode and frequency of administration [2], chance of a beneficial effect [3], risks, and [4] follow-up (see Table 1).

**Table 1:** Attributes and levels included in the DCE

Attribute	Level	Explanation for the participant
Mode and frequency of administration		The way the drug enters the body and how many times the drug should be given.
	Single operation *	You will be under general anesthesia (in a deep sleep) during the operation. The operation is one-time with a permanent effect. The drug is introduced into the brain through an injection.
	Lumbar puncture 12 times per year	A lumbar puncture is an injection in the lower back. During this treatment you are awake and the skin can be made numb locally. A lumbar puncture has a temporary effect and must therefore be repeated every month.
	Lumbar puncture 6 times per year	A lumbar puncture is an injection in the lower back. During this treatment you are awake and the skin can be made numb locally. A lumbar puncture has a temporary effect and must therefore be repeated every two months.
Chance of a beneficial effect		The number of people that experience a good result, such as slowing down disease progression. The exact chance is currently not known, therefore this chance is hypothetical.
	20%	20 in 100 persons experienced a good result.
	40%	40 in 100 persons experienced a good result.
	60%	60 in 100 persons experienced a good result.
Risks		The percentage of people that experience a negative side effect.
	1% risk of infection, bleeding, paralysis *	Short-term side effects that can arise immediately after the treatment. There is a 1% risk (1 in 100 persons) of side effects such as infection, bleeding, or even paralysis or death. These side effects can cause permanent damage.
	10% risk of headache, pain at injection site	Short-term side effects that can arise immediately after the treatment. There is a 10% (10 in 100 persons) risk of side effects such as headache or pain on the injection site. These side effects will pass.
	Unknown on long-term	Long-term side effects that occur later, for example after years. The long-term side effects of genetic interventions are currently not known. It is also not known how likely these are to occur. Possible risks that can occur are for example undesirable effects of the injection of genetic material into the brain.
Follow-up		The healthcare provider and hospital that will conduct the follow-up appointments during the treatment period. Please note: this question is about follow-up appointments. A possible operation will always take place in the nationwide expert center for SCA or HD.
	Neurologist in local hospital without expertise *	A neurologist who does not have specific knowledge of SCA or HD, working in the nearest local hospital.
	Neurologist in nearest university hospital without expertise	A neurologist who does not have specific knowledge of SCA or HD, working in the nearest university hospital.
	Neurologist in nationwide expert center (University hospital)	A neurologist who is familiar with SCA or HD, working in the nationwide expert center for SCA or HD. This is a university hospital.
	Nurse practitioner in nationwide expert center (University hospital)	A nurse practitioner who is familiar with SCA or HD, working in the nationwide expert center for SCA or HD. This is a university hospital.

^*^ Level of the attribute which was used as the reference level for dummy coding

### Selection of the levels

For each attribute, levels and their descriptions were selected based on a review of the literature, information from ongoing clinical trials and from websites of pharmaceutical companies that are developing genetic interventions for SCA and HD. Since genetic interventions are not available yet for patients with SCA and HD, some levels were estimated based on the results of the semi-structured interviews and expert opinion of team members. Following the ISPOR guidelines, we did not use ranges to define attributes and we limited levels to three or four per attribute.

Levels for the attribute ‘mode and frequency of administration’ were chosen based on genetic interventions that are currently being studied [6]. Levels for the attribute ‘chance of a beneficial effect’ were chosen based on expert opinion and assumptions that came forward during the interviews with patients, where inclusion of outliers (i.e. extreme, unrealistic values) was avoided. Levels for the attribute ‘risks’ were chosen based on known side effects of lumbar punctures and intracerebral injections [26], and possible long-term effects were based on expert opinion. Levels of the attribute ‘follow-up’ were chosen based on logistic options within the healthcare system in the Netherlands (see Table 1).

### Experimental design and questionnaire

Based on the number of attributes and levels, there are 3^3^ × 4^1^ = 108 hypothetical treatment combinations. For practical reasons, *Ngene* (version 1.1.1. http://www.choice-metrics.com) was used to reduce this number to a manageable size by the development of an Bayesian efficient experimental design with 24 choice sets divided into three blocks of eight. Blocking was applied to reduce the number of tasks per respondent and thus cognitive burden.

Full profiles were used which means that within each task, a respondent was presented all attributes that were included in the study. Profiles were grouped into sets of two per task (see Table 2). Respondents were randomly assigned to one of the three blocks, based on registration number.

The forced choice-elicitation format was used. No opt-out or status-quo options were included, since no good alternative treatment is currently available for patients with SCA and HD.

Each respondent was also given an additional choice set that checked for internal validity. This task included a within-set dominated pair (i.e. a choice set with alternative A is more desirable than alternative B for all attributes) [27], to check whether the respondents choose the dominated alternative within the choice set. A sensitivity analysis was done to check for the effect of excluding this choice set from the analysis.

To improve the readability of the questionnaire, the text was screened and adapted by a communication expert of Radboud university medical center. A pilot test of the DCE was conducted in 19 participants with SCA or HD, to check whether respondents understood the choice sets and explanations. All 10 participants who previously participated in the semi-structured interviews, were asked to fill in the pilot questionnaire, as well as nine members of the Dutch patient association for ataxia. The questionnaires of the pilot were analyzed and these results were used to update the Bayesian efficient design.

The choice tasks were part of an online structured questionnaire that also included additional open ended- and multiple choice questions, such as questions about health status, sociodemographic information, and some contextual questions related to the choice tasks. The additional questions are listed in Supplement 1. The questionnaire was built in the web-based survey tool LimeSurvey.

Prior to the actual choice tasks, the questionnaire included a simple example question, in order to introduce the concepts of ‘attributes’ and ‘levels’ to the respondents. Furthermore, additional information, descriptions and explanations about the used attributes and levels was provided prior to the choice tasks and all participants had the option to read the explanation again at the moment the choice sets were presented.

At the end of the choice sets, participants were asked how clear the questions were on a 5-point scale ranging from ‘very clear’ to ‘very unclear’, and how difficult it was for them to choose between the treatments in the choice sets, also on a 5-point scale ranging from ‘very easy’ to ‘very difficult’.

**Table 2:** Example of a choice set in the DCE

Characteristics	TREATMENT A	TREATMENT B
***Mode and frequency of administration***	Lumbar puncture 6 times per year	Single operation
***Chance of beneficial effect***	20% (20 in 100 persons experienced a good result)	20% (20 in 100 persons experienced a good result)
***Risks***	10% risk transient side effects as headache, pain at injection site	Unknown on long-term
***Follow-up***	Nurse practitioner in nationwide expert center (university hospital)	Neurologist in nearest university hospital without expertise

### Data collection

Data were collected between April 2022 and January 2023, with help of the Dutch patient associations for ataxia and HD. Adult patients with a confirmed diagnosis of SCA, HD or persons who carry a pathogenic CAG repeat expansion in a SCA or HD disease-causing gene were invited to participate. Respondents were recruited to complete the online questionnaire by placing a call with a link to the questionnaire on the patient organizations’ online media platforms, such as their websites, Facebook pages, and digital newsletters. Patients were sent a paper version of the questionnaire on request.

All respondents were asked to give consent for the use of their anonymous responses before the questionnaire started.

The Regional Ethics Committee Arnhem-Nijmegen, the Netherlands concluded that the Medical Research Involving Human Subjects Act (WMO) did not apply to this study (file number: 2021–9700).

Based on the number of active members of the Dutch patient associations for SCA and HD, and based on prior respondent rates within these groups, it was estimated that it would be feasible to include 300 respondents, which is similar to the number that is recommended by others for robust quantitative analysis [28].

### Statistical analysis

Descriptive data were analyzed with SPSS version 27 for Windows. The independent sample’s T test was used to compare means between two groups. Spearman’s rank correlation test was used to test whether ordinal variables correlated. A significance level of 0.05 was chosen for statistical significance.

Discrete choice data were analyzed using *Nlogit* version 5 (Econometric Software, Inc.).

A multinominal logit (MNL) model was used to estimate the effect of the attribute levels on preferences of the respondents. The four attributes were modeled as determinants for the decision for ‘treatment A’ or ‘treatment B’. The regression equation for this model is:

*U*_i_ = β_0_ + β1 * lumbar puncture 6 times a year + β2 * lumbar puncture 12 times a year + β3 * beneficial effect + β4 * risk of 10% + β5 * unknown risk + β6 * follow-up nearest university hospital + β7 * follow-up nurse expert center + β8 * follow-up neurologist expert center + ε_i_.

Whereas *U* is the relative utility for a genetic intervention A or B, β0 is the constant, β1 to β8 are the specific attribute utility weights, and ε is an unobserved component or the error.

The attribute levels of the attributes ‘mode and frequency of administration’, ‘risks’ and ‘follow-up’ were categorical variables and therefore, dummy coding was applied. ‘Single operation’, ‘risk of 1%’ and ‘follow-up in nearest local hospital’ were used as reference levels for the abovementioned attributes (see Table 1). ‘Chance of a beneficial effect’ was considered a continuous variable. A positive or negative sign indicates if a level is preferred or not preferred over the reference level. For the attribute ‘chance of a beneficial effect’, a positive sign was expected but for the other attributes, no a priori hypothesis was formulated.

Model fit was assessed using log likelihood and McFadden’s pseudo R^2^. A constant term was included to check for left-to-right bias, which is a marker for a tendency to choose the first option in the choice task.

To explore if preferences for specific attributes depend on the underlying disease (HD or SCA), interactions were added to the model. In addition, subgroup analyses were performed with the co-variate disease severity to examine if severity influenced preferences in these subgroups. To check for reliability of the results of the main model, a sensitivity analysis was conducted excluding the results of the choice set that checked for internal validity.

Two subcategories for disease severity were established. SCA patients with no symptoms or who could still walk independently, and HD patients with disease stage 1 were classified as ‘mild’. SCA patients who needed a walking aid or wheelchair, and HD patients with disease stages 2 or higher were classified as ‘severe’.

## Results

### Respondents characteristics

The online questionnaire was started by 289 persons and completed by 214. Two respondents were excluded because they did not give informed consent for the use of their responses. Furthermore, four persons filled in a paper version of the questionnaire. In total, the results of 216 respondents were included. Characteristics of the respondents are summarized in Table 3.

**Table 3:** Respondent characteristics

	*N*	%
**Sex**		
Male	108 / 216	50%
Female	108 / 216	50%
**Disease**		
SCA	115 / 216	53.2%
HD	89 / 216	41.2%
Other ^1^	12 / 216	5.6%
**SCA subtype (** ***n*** ** = 115)**		
SCA1	9 / 115	7.8%
SCA2	1 / 115	0.9%
SCA3	58 / 115	50.4%
SCA6	26 / 115	22.6%
SCA7	1 / 115	0.9%
SCA 8, 10, 12 or 36	2 / 115	1.7%
Idiopathic late onset cerebellar ataxia	1 / 115	0.9%
Other autosomal dominant cerebellar ataxia	9/ 115	7.8%
Autosomal recessive cerebellar ataxia	0 / 115	0%
No genetic testing	3 / 115	2.6%
Other	5 / 115	4.3%
**SCA (and ‘others’) level of functioning**^**1**^ [38] (***n* = 127)**		
No symptoms	15 / 127	11.8%
Symptoms, walk independent	47 / 127	37%
Symptoms, walk with walking aid	49 / 127	38.6%
Symptoms, wheelchair bound	16 / 127	12.6%
**HD level of functioning**^**1**^ [39] (*n*** = 89)**		
Stage 1 (TFC-UHDRS^2^ score 11–13)	49 / 89	55.1%
Stage 2 (TFC-UHDRS score 7–10)	30 / 89	33.7%
Stage 3 (TFC-UHDRS score 3–6)	10 / 89	11.2%
Stage 4 (TFC-UHDRS score 1–2)	0 / 89	0%
Stage 5 (severe disability)	0 / 89	0%
**Living situation**		
Single	43 / 216	19.9%
With child(ren)	5 / 216	2.3%
With partner	107 / 216	49.5%
With partner and child(ren)	58 / 216	26.9%
With parents	2 / 216	0.9%
Nursing home	1 / 216	0.5%
**Highest level of education**		
Basic (ISCED-11^3^ level 1–2)	31 / 216	14.4%
Intermediate (ISCED-11 level 3–4)	78 / 216	36.1%
Advanced (ISCED-11 level 5–8)	107 / 216	49.5%
**Most disabling symptom (SCA) (** ***n*** ** = 115)**		
Movement / coordination / walking	81 / 115	70.4%
Speech	15 / 115	13%
Fatigue / energy	10 / 115	8.7%
Mood (depression)	1 / 115	0.9%
Other	8 / 115	7%
**Most disabling symptom (HD) (** ***n*** ** = 89)**		
Movement / coordination / walking / chorea	28 / 89	31.5%
Speech / swallowing	7 / 89	7.9%
Memory / cognition	15 / 89	16.9%
Behavioral changes	22 / 89	24.7%
Mood (depression)	5 / 89	5.6%
Other	12 / 89	13.5%
**Ideal timing of genetic intervention**		
Before first symptoms	64 / 216	29.6%
First symptoms	105 / 216	48.6%
Need for walking aid	25 / 216	11.6%
Unable to do job	8 / 216	3.7%
Need for nursing home	7 / 216	3.2%
Other	7 / 216	3.2%
**Wants to participate in trial**		
Yes	173 / 216	80.1%
No	43 / 216	19.9%
**Reason to participate in trial (** ***n*** ** = 173)**		
Earlier timing to receive treatment	43 / 173	24.9%
Contribution to science	28 / 173	16.2%
For future generations (children)	92 / 173	53.2%
No costs	0 / 173	0%
More follow-up	6 / 173	3.5%
Other	4 / 173	2.3%
**Reason not to participate in trial (** ***n*** ** = 43)**		
Unknown risks	26 / 43	60.5%
Chance of receiving placebo	3 / 43	7%
Time	2 / 43	4.7%
Travelling	6 / 43	14%
Other	6 / 43	14%

^1^ For determination of the level of functioning, these 12 respondents were added to the ‘SCA’ subgroup

^2^ TFC-UHDRS = Total Functional Capacity of the Unified Huntington Disease Rating Scale

^3^ ISCED-11 = International Standard Classification of Education 20

Mean age of the respondents was 54.7 years (range 22–86 years, 2 with missing values). Twelve respondents filled in ‘other’ to the question which disease they had, however, of those 12 respondents, seven had a form of ataxia and one had premanifest HD. Three respondents had a genetic form of ataxia in their families and were asymptomatic, and one respondent had further unspecified ‘neurological symptoms’. As all but one had appropriate symptoms or a positive family history, we decided to not exclude this small group of respondents from the final analysis.

The most common subtype of SCA was SCA3 (58 respondents) followed by SCA6 (26 respondents).

A total of 111 respondents were categorized in a mild disease stage, and 105 respondents were categorized in a severe disease stage.

For respondents with SCA and HD, the most disabling symptom was the problem with movement and coordination (*n* = 81 for SCA and *n* = 28 for HD), followed by behavioral changes in respondents with HD (*n* = 22).

Out of 216 patients, 198 (91.7%) had a travelling time of two hours or less to the nearest nationwide expert center for SCA or HD.

### Opinions on timing and trials

When respondents were asked what the ideal timing to start a genetic intervention was, most indicated that it would be the moment the first symptoms arise (*n* = 105; 48.6%), followed by starting treatment in the premanifest disease stage (*n* = 64; 29.6%) (see Table 3). For respondents with SCA, the current disease stage positively correlated with the ideal timing of treatment, meaning that patients with a more advanced disease stage preferred a later start of genetic intervention as compared to patients with a less severe or premanifest disease stage (correlation coefficient 0.299; *p* < 0.001). For patients with HD, there was a similar trend but without statistical significance (*p* = 0.10).

80% of respondents (*n* = 173) were willing to participate in a trial with genetic interventions. The most important reason for this decision was to gain knowledge for future generations (*n* = 92). Only 43 respondents said they would not participate in a trial with genetic interventions, mainly because of unknown risks (*n* = 26) (see Table 3).

### Feasibility of the DCE

On the question ‘how clear did you find the questions where you had to make a choice between the two treatments?’, most patients indicated that the choice sets were clear to read (score ‘very clear’ or ‘clear’; *n* = 193; 89.3%). However, on the question ‘did you find it difficult to make a choice between the two treatments or was it easy to choose for you?’ only 15.3% said the decisions were difficult to make, while 40.7% felt this was not easy/not difficult. 43% of respondents found it easy to make a decision (score ‘very easy’ and ‘easy’ *n* = 93).

### DCE choice task

Results of the main effect model are shown in Table 4.

**Table 4:** Attribute preferences for all respondents and specified by disease

	All respondents			SCA			HD		
	β-coefficient	Significance	95%CI	β-coefficient	significance	95%CI	β-coefficient	significance	95%CI
Constant	0.032	NS	-0.087; 0.152	-0.080	NS	-0.233; 0.073	0.211	0.05	0.014; 0.408
***Mode and frequency of administration***									
Single operation	Reference level								
Lumbar puncture 6 times a year	-0.702	0.01	-0.871; -0.532	− 0.735	0.01	-0.954; -0.516	-0.675	0.01	-0.951; -0.400
Lumbar puncture 12 times a year	-1.429	0.01	-1.606; -1.252	-1.358	0.01	-1.585; -1.130	-1.599	0.01	-1.893; -1.304
***Chance of beneficial effect***									
	0.072	0.01	0.064; 0.080	0.068	0.01	0.058; 0.078	0.079	0.01	0.066; 0.093
***Risks***									
1% risk	Reference level								
10% risk	-0.028	NS	-0.195; 0.139	0.067	NS	-0.147; 0.80	-0.169	NS	-0.444; 0.106
Unknown long-term risk	-0.089	NS	-0.251; 0.072	0.119	NS	-0.090; 0.328	-0.420	0.01	-0.683; -0.157
***Follow-up***									
Nearest local hospital	Reference level								
Nearest university hospital	-0.031	NS	-0.248; 0.185	0.004	NS	-0.272; 0.279	-0.084	NS	-0.442; 0.274
Nurse expert center	0.164	NS	-0.040; 0.369	0.196	NS	-0.066; 0.458	-0.100	NS	-0.235; 0.435
Neurologist expert center	0.043	NS	-0.176; 0.261	0.112	NS	-0.174; 0.397	-0.068	NS	-0.414; 0.277
Log Likelihood	-900.062			-542.891			-346.457		
Chi squared	874.219	0.000		464.748	0.000		427.593	0.000	
Adjusted pseudo R^2^	0.32								
AIC/N	0.935			0.973			0.878		
Number of responders*	216			126 *			90		
Number of observations	1944			1134			810		

* The 12 respondents who filled in ‘other underlying disease’ were added to the SCA subgroup for statistical analysis, except for the premanifest HD patient

NS = not significant

Two attributes, ‘mode and frequency of administration’ and ‘chance of a beneficial effect’ had a statistically significant impact on the respondents’ preference for a genetic intervention (*p* = 0.01). The attributes risks and follow-up were not of statistical significant influence on the decision of the total group of respondents.

Respondents preferred lumbar punctures 6 and 12 times a year less compared to a single operation, given the negative β-coefficients of these two attribute levels (-0.702 and − 1.429, respectively, both with *p* = 0.01). As expected, respondents preferred a higher chance of a beneficial effect (coeffient: 0.072, *p* = 0.01).

For the total group, the constant coefficient was not significant, meaning that there was no left to right bias in choosing the left versus right options.

### Differences between subgroups

The interaction model showed that there was statistical significant interaction between the attribute level ‘unknown long-term risk’ (coefficient − 0.545; *p* = 0.01) and the covariate ‘underlying disorder’; meaning that the preference for this level of the attribute ‘risks’ depended on having SCA or HD as underlying disorder. The interaction model slightly improved model fit (likelihood ratio − 894.432 with pseudo R^2^ 0.33), and showed that respondents with HD statistical significantly prefer a 1% risk of possible permanent side-effects over unknown long-term risks (results not shown in tables).

When performing an explorative analysis within the subgroups SCA and HD, adding the covariate disease severity to the model, it was shown that SCA patients with a mild disease severity prefer repeated lumbar punctures less compared to a single operation, a higher beneficial effect, and a 10% risk of transient side-effect over a 1% risk of possible permanent side-effects. Furthermore, they prefer follow-up with a nurse practitioner in an expert center over follow-up with a neurologist in the nearest local hospital. Patients with HD with mild disease severity prefer repeated lumbar punctures less compared to a single operation and a higher beneficial effect.

Both SCA and HD patients in a severe disease stage preferred repeated lumbar punctures less compared to a single operation and a higher beneficial effect. In addition, HD patients with severe disease prefer a 1% risk of possible permanent side-effects over an unknown long-term risk. (see Table 5).

**Table 5:** Attribute preferences, specified by disease and disease severity

	SCA – mild			SCA – severe			HD – mild			HD – severe		
	β-coefficient	significance	95%CI	β-coefficient	significance	95%CI	β-coefficient	significance	95%CI	β-coefficient	significance	95%CI
Constant	-0.390	NS	-0.278; 0.200	-0.120	NS	-0.325; 0.084	0.018	NS	-0.246; 0.281	0.459	0.01	0.149; 0.769
***Mode and frequency of administration***												
Single operation	Reference level											
Lumbar puncture 6 times a year	-0.860	0.01	-1.203; -0.517	-0.687	0.01	-0.976; -0.398	-0.618	0.01	-0.982; -0.253	-0.759	0.01	-1.192; -0.325
Lumbar puncture 12 times a year	-1.610	0.01	-1.984; -1.235	-1.282	0.01	-1.582; -0.982	-1.481	0.01	-1.872; -1.089	-1.801	0.01	-2.267; -1.336
***Chance of beneficial effect***												
	0.091	0.01	0.073; 0.109	0.055	0.01	0.042; 0.068	0.085	0.01	0.067; 0.103	0.074	0.01	0.052; 0.096
***Risks***												
1% risk	Reference level											
10% risk	0.340	0.05	0.000; 0.679	-0.070	NS	-0.357; -0.216	-0.076	NS	-0.443; 0.291	-0.309	NS	-0.746; 0.129
Unknown long-term risk	0.267	NS	-0.056; 0.590	0.050	NS	-0.230; 0.329	-0.278	NS	-0.625; 0.070	-0.623	0.01	-1.038; -0.209
***Follow-up***												
Nearest local hospital	Reference level											
Nearest university hospital	0.339	NS	-0.129; 0.806	-0.184	NS	-0.539; 0.171	-0.273	NS	-0.753; 0.206	0.116	NS	-0.436; 0.668
Nurse expert center	0.472	0.05	0.057; 0.886	0.017	NS	-0.333; 0.367	-0.121	NS	-0.570; 0.328	0.380	NS	-1.150; 0.909
Neurologist expert center	0.300	NS	-0.146; 0.746	-0.006	NS	-0.397; 0.385	-0.370	NS	-0.844; 0.104	0.284	NS	-0.240; 0.807
Log Likelihood	-240.751			-297.604			-191.555			-147.709		
Chi squared	282.208	0.000		203.959	0.000		235.156	0.000		203.547	0.000	
AIC/N	0.895			1.048			0.891			0.871		
Number of respondents *	62			65			49			40		
Number of observations	558			585			450			360		

* The 12 respondents who filled in ‘other underlying disease’ were added to the SCA subgroup for statistical analysis (for these patients the level of functioning was based on ref. Klockgether et al.)

NS = not significant

For respondents with HD, and especially those with severe symptoms, a strong left to right bias is seen, given the significance of the constants of 0.211 and 0.459, with p-values of 0.05 and 0.01, respectively, which indicates that they often chose the first (left) option. Additional analyses indeed showed that respondents with HD statistical significantly choose option A more frequently as compared to respondents with SCA (*p* = 0.012).

### Reliability

Nine out of 216 respondents (4.2%) choose the non-dominant option in the choice set that tested for internal validity (i.e. the option that was less desirable than its alternative for all attributes). The other respondents (95.8%) choose the option the researchers would expect, namely the alternative with the best levels.

### Sensitivity analysis

A sensitivity analysis excluding responses to the dominant choice set for all respondents showed no difference in the significance of the results of the main effect model (see Supplement 1). The likelihood ratio slightly improved to -854.612 with an adjusted pseudo R^2^ of 0.36. In the interaction model, the significance level of ‘10% risk of transient side effects’ changed from 10–5%. In the subgroup analysis the constant in the model for patients with HD was not significant anymore.

## Discussion

In this study, a DCE quantified the preferences of patients with SCA or HD regarding genetic interventions. The results of this DCE show that mode and frequency of administration and chance of a beneficial effect influence the choice for a genetic intervention in patients with manifest or premanifest SCA or HD, and that preferences for certain attributes depend on the underlying disease and disease stage.

This study includes the largest group of respondents in a DCE regarding genetic interventions to date, and is the first to get insight into the preferences of patients with SCA and HD. In 2021, Witkop et al. published the results of a DCE on preferences for gene therapy in patients with haemophilia. Interestingly, the haemophilia patients found the beneficial effect on bleeding rate the most important attribute, followed by dose frequency and durability [29], which is comparable to the results of this study. A recent DCE among patients with spinal muscular atrophy and their caregivers showed that improvement in current functioning was highly valued, and that oral medication and one-time infusion was strongly preferred over repeated intrathecal injections [30]. In conclusion, all these DCE’s show that attributes reflecting the clinical effect and the administration process are considered important by patients.

Two previous studies used a survey to obtain insight into SCA and HD patients’ preferences regarding genetic interventions in the hypothetical context of a clinical trial [21, 22]. These qualitative studies showed that patients are willing to undergo genetic interventions. For patients with ataxia, the potential benefit is a common motivation to participate in a trial with genetic interventions and 70% of patients were willing to undergo intrathecal drug administration [22]. These results seem to be in line with the results of the current study, as beneficial effect influenced the decision for a certain genetic treatment, and a single operation was preferred over repeated lumbar punctures. In the previous study with patients with HD, it was concluded that patients preferred treatments that are less invasive and need to be administered less frequently [21]. The likelihood of patients with HD to enroll in a trial lowered in scenarios with more invasive surgical interventions [21], while respondents in the current study preferred a single operation over repeated lumbar punctures. Possibly, the contextual difference (clinical trial versus actual care) might play a role here.

Interestingly, the ideal timing for most patients was the moment the first symptoms arise, and not before the start of first symptoms. This is of major relevance as the HD and SCA field focus on the identification of premanifest carriers who are close to disease conversion – this stage is regarded as the optimal treatment window given that neuronal cell decline starts before the first clinical symptoms emerge [31, 32]. However, it is too preliminary to conclude on a possible divergence of patient versus academic perspective, as the ideal timing to start genetic interventions correlated with disease severity. Patients with a more severe disease stage preferred a later start compared to patients with a less severe or premanifest disease stage. This finding can result from a shift in the disease stage that is acceptable for someone with a disease, a phenomenon called ‘response shift’, which was also was observed in the semi-structured interviews.

### Limitations

In general, this study included a complex DCE as this DCE included hypothetical scenarios and attributes with combined characteristics (i.e. the attribute ‘risks’ included a percentage and examples of side effects, the attribute ‘follow-up’ included a location and a level of expertise). Nevertheless, most respondents indicated they found the questions clear to read and not difficult to make a choice.

The level of education of the respondents was high, as compared to the mean highest achieved level of education of the general population in the Netherlands, which is ISCED 3–4 [33]. The web-based recruitment of respondents may have led to inclusion bias, as more highly educated and mainly mildly affected individuals responded. These persons might have better access to, and might be more active on the online platforms of patient associations. Furthermore, it is known that the cognitive burden of a DCE can reduce response rates [34], and this might be an additional explanation for a relative small proportion of lower educated and more severely affected respondents. Therefore, one should be cautious to extrapolate the results to the entire population of patients with SCA and HD. In order to broaden generalizability in future studies it could be helpful to send individuals a paper version of the questionnaire, and to offer help with completing the questionnaire.

A high percentage of respondents indicated that they were willing to participate in a trial with genetic interventions. Selection bias might have also played a role here, as the respondents to our questionnaire are active on the online platforms of patient associations, a place where trials for new treatments are followed closely.

In order to minimize the cognitive burden of the DCE, we minimized the number of attributes to four and the number of choice sets to eight. Nevertheless, the results show that patients with HD, and in particular patients with severe HD, tended to always choose the left alternative as the constant was significant. Normally, in an unlabeled design, the constant should be nonsignificant to assume that patients are considering all information in both alternatives and then choose the one that gives the highest utility. However, a significant constant indicates that respondents pay more attention to the information presented on the left and more often choose that alternative [35]. The left-to-right bias might be due to the cognitive burden experienced by patients with HD, in particular those with severe symptoms, and should be kept in mind when designing future surveys or DCEs for this population.

The fact that some of the results were non-significant suggested that these attributes were not relevant for the respondents’ decisions. Interestingly, the two attributes that always did reach significance were placed in the upper two rows of each choice task, and the nonsignificant attributes in the lowest two rows. For practical reasons and for the readability of the choice sets, randomization of the order of attributes was not applied. Possibly, the fact that people read from top to bottom may be of influence if respondents did not fully read the full choice task before making a choice: a phenomenon known as top-to-bottom bias [35]. An explanation for the non-significance of the attribute ‘risk’ in the main analysis could be that this attribute was difficult to interpret for respondents as it included a combination of a chance and a certain severity; respondents could have ignored this attribute in their decision making.

Because genetic interventions for SCA and HD are currently in the preclinical and first clinical trial phases, some of the chosen levels for the attributes ‘chance of a beneficial effect’ and ‘risk’ where chosen by the research team and not yet evidence-based. The results of clinical trials might show that chances of effect and risks might be different than here assumed, and this could subsequently result in other preferences and decisions of patients.

96% of respondents choose the dominant option in the choice task that checked for internal validity. For the analysis, we decided to include the responses of the respondents who choose the non-dominant option to this choice task, as deleting responses can result in the removal of valid preferences [36]. In addition, it can be questionable whether the ‘’irrational’’ responses of nine respondents who chose the non-dominant option were truly irrational or only deemed by the researchers to be worse. In that case, the learning process of the respondent or shortcomings in the study design can be held accountable for their choice [36]. Overall, we decided to include the internal validity test in our main analysis because we considered the information from this choice sets still informative. However, we additionally performed sensitivity analysis to examine if results without the within dominance choice set were comparable, which turned out to be the case.

Although the aimed number of 300 respondents was not reached, the number of 216 respondents can be considered sufficient for the analyses that were conducted. Unfortunately, there is no formal guidance on estimating the optimal sample size for DCE data although Louviere and Lancsar mention that one rarely requires more than 20 respondents per version to estimate reliable models [37]. This means that for our study a minimum sample size of 60 respondents (3 versions of the questionnaire* 20 respondents) would be required. Taking into account that we also performed subgroup analysis, the number of 216 respondents can be considered sufficient.

### Implications for further scientific and clinical developments

The results of this study may contribute to the design of future trials and future clinical care pathways. For example, patients seem to prefer a single surgical procedure over repeated lumbar punctures. This would require academia an industry to prioritize non-ASO interventions and/or alternative delivery. However, in the semi-structured interviews that were conducted in preparation of the DCE, patients who did not have had a lumbar puncture before seemed to be more hesitant than patients who did. Adequate information on lumbar punctures may improve the willingness to undergo these. Oral administration was not added as a treatment option in the current DCE. However, oral administration of genetic therapy is also being tested: A recent study on Branaplam (i.e. VIBRANT-HD; NCT05111249) was ended prematurely because of side effects.

Unexpectedly, expertise did not seem to play a large role in the decision-making process as patients, except for the group with mild SCA, did not significantly prefer follow-up in an expert center over follow-up in the local hospital. In local hospitals, healthcare professionals generally do not have specific expertise with rare hereditary movement disorders. We have not explored reasons for this preference of local hospital versus expert center. Patients with mild SCA did prefer follow-up in an expert center, which may be due to their better physical possibilities, as compared to patients with more severe stages of SCA. Translating this into clinical practice, future care pathways might be organized in a way that patients, especially those in more advanced disease stages, can receive follow-up to further monitor the effect of new invasive genetic treatments in a local hospital.

## Conclusion

This study shows how, in a scientifically sound way, patient involvement can be used to provide insight into patient perspectives on genetic interventions, which can contribute to shaping of patient-centered healthcare. study shows that the frequency and mode of administration, and the chance of a beneficial effect are both of influence on the decision for a certain genetic intervention in patients with SCA and HD. Patients prefer repeated lumbar punctures (6 times or 12 times yearly) less compared to a single operation. The scientific versus patient perspective on the ideal timing of genetic interventions requires further study. These results provide guidance to design upcoming clinical trials and, if proven effective, future implementation of genetic interventions in patient-centered healthcare pathways.

## Electronic supplementary material

Below is the link to the electronic supplementary material.

Supplementary Material 1

Supplementary Material 2

## Data Availability

The data that support the findings of this study are not openly available due to reasons of sensitivity and are available from the corresponding author upon reasonable request. Data are located in controlled access data storage at Radboud university medical center.
