# Supplementary material for: Preferences for genetic interventions for SCA and Huntington’s disease: results of a discrete choice experiment among patients
Source: Orphanet J Rare Dis. 2024 Oct 28;19:398. doi: 10.1186/s13023-024-03408-2 (PMC11514962; doi:10.1186/s13023-024-03408-2)
Supplement: Supplementary file 1 — Supplementary Material 1 [file 13023_2024_3408_MOESM1_ESM.docx]

**Supplement 1:** Additional questions in the questionnaire

**General questions**

1. **What is your age?**

_________years

1. **What is your gender?**

- Female
- Male

1. **Which disorder do you have?**

- Spinocerebellar ataxia (SCA) *🡪 go to question 4*
- Huntington’s disease *🡪 go to question 6*
- Other *🡪 go to question 4*

1. **Which type of ataxia do you have?**

- SCA1
- SCA2
- SCA3
- SCA6
- SCA7
- SCA17
- SCA8, SCA10, SCA12 or SCA36
- Another form of SCA or ADCA (autosomal dominant cerebellar ataxia)
- ARCA (autosomal recessive cerebellar ataxia) or a subtype such as Friedreich’s ataxia or ataxia telangiectasia
- ILOCA (ideopathic late onset cerebellar ataxia) of MSA-C (the cerebellar form of multi system atrophy)
- I do not have a genetic diagnosis / I never was genetically tested
- Other

1. **What is you functioning level at this moment?**

- I do not have symptoms
- I have symptoms but I can walk independently
- I can walk independently with help from others or with a walking aid
- I cannot walk and I use a wheelchair
- *Ga to question 11 (you can skip questions 6 to 10)*

1. **This question is about your functioning level at this moment.
   Are you able to work?**

- Yes (normal occupation)
- I have reduced capacity for my usual job
- I can do marginal work only
- I am unable to work

1. **This question is about your functioning level at this moment.
   Are you able to do your financial tasks yourself?**

- Yes
- With slight assistance
- With major assistance
- I am unable to do my financial tasks myself, someone helps me.

1. **This question is about your functioning level at this moment.
   Are you able to do domestic chores?**

- Yes
- I am impaired
- No

1. **This question is about your functioning level at this moment.
   Are you independent for your activities of daily living?**

- Yes
- I am minimal impaired
- I can do gross tasks only
- I need total care

1. **This question is about your functioning level at this moment.
   What is your living situation?**

- I live at home without care
- I live at home and receive chronic care
- I live in a nursing home and need full time skilled nursing

1. **What is your living situation?**

- I live alone
- I live with my partner
- I live with my partner and child(ren)
- Other

1. **What is your highest level of education?**

- ISCED 0: No education
- ISCED 1: Primary education (primary school)
- ISCED 2: Lower secondary education
- ISCED 3: Upper secondary education
- ISCED 4: Post-secondary non-tertiary education
- ISCED 5: Short-cycle tertiary education
- ISCED 6: Vocational education (Bachelor’s)
- ISCED 7: University education (Master)
- Other

**Additional questions after the choice sets:**

1. **What is your travelling time (single journey) to the nearest nationwide expert center for SCA/HD?**

- Less than 30 minutes single journey
- 30 to 60 minutes single journey
- One to two hours single journey
- More than two hours single journey

*Dutch nationwide expert centers for ataxia are located in Groningen (UMCG) and Nijmegen (Radboudumc). Dutch nationwide expert centers for Huntington’s disease are located in Groningen (UMCG), Leiden (LUMC) and Maastricht (MUMC+).*

1. **Which most disabling symptom do you hope to see improved by the treatment?**

   In case you have Huntington’s disease:

- Movement / walking / coordination / chorea
- Speech / swallowing
- Memory / cognition
- Changes in my behavior
- Mood (depression)
- Other

In case you have ataxia:

- Movement / walking / coordination
- Speech
- Fatigue / energy
- Mood (depression)
- Other

*Please choose the most disabling symptom, also in case you have more than one symptom.*

1. **If you would have the choice, at what moment in your disease course would you prefer to be treated?**

- When I do not have symptoms yet
- When the first symptoms emerge
- When I need walking aids due to the symptoms
- When I am unable to do my job due to the symptoms
- When I am unable to live in my home due to the symptoms
- Other

*Because this question is hypothetical, you can also choose an earlier disease stage than the stage you are in now.*

1. **Would you participate in a clinical trial with genetic interventions (i.e. would you undergo genetic interventions for your disease when this treatment is not yet proven effective but when it is tested in a clinical trial?)**

- Yes *🡪 go to question 17 (and skip question 18)*
- No *🡪 go to question 18*

*If a treatment is studied in a clinical trial, this means that the effect and safety of the treatment is tested in a small group of people. The treatment is not yet available for every patient at that time. Participation in a trial has advantages and disadvantages.*

1. **You have answered that you do want to participate in a study in which a new genetic intervention is being tested. What is your most important argument for this?**

- Because I will receive the treatment sooner than when I need to wait for the studies to be finished.
- Because I want to make a contribution to science.
- Because I find it important for my children (or other family members) that this future treatment is being investigated.
- Because I don’t have to pay for the treatment
- Because I will have more frequent follow-up at the hospital and my doctors will keep a closer eye on me.
- Other

*Please choose your most important reason in case you have more than one.*

1. **You have answered that you do not want to participate in a study in which a new genetic intervention is being tested. What is your most important argument for this?**

- Because the risks and side effects are not known.
- Because there is a possibility that I will receive a placebo (this is a ‘’fake’’ drug)
- Because participating in a study will take too much of my time
- Because travelling to the treatment center is difficult for me
- Other

*Please choose your most important reason in case you have more than one.*

**Evaluation**

1. **How clear did you find the questions where you had to make a choice between the two treatments?**

- Very clear
- Clear
- Not clear / not unclear
- Unclear
- Very unclear

1. **Did you find it difficult to make a choice between the two treatments or was it easy to choose for you?**

For me it was…

- Very easy
- Easy
- Not easy / not difficult
- Difficult
- Very difficult
