# Supplementary material for: Preferences for genetic interventions for SCA and Huntington’s disease: results of a discrete choice experiment among patients
Source: Orphanet J Rare Dis. 2024 Oct 28;19:398. doi: 10.1186/s13023-024-03408-2 (PMC11514962; doi:10.1186/s13023-024-03408-2)
Supplement: Supplementary file 2 — Supplementary Material 2 [file 13023_2024_3408_MOESM2_ESM.docx]

**Supplement 2.** Analyses excluding the results of the choice set with a dominant option.

|  | **All respondents** | | | **SCA** | | | **HD** | | |
| --- | --- | --- | --- | --- | --- | --- | --- | --- | --- |
|  | β-coefficient | significance | 95%CI | β-coefficient | significance | 95%CI | β-coefficient | significance | 95%CI |
| Constant | 0.011 | NS | -0.112; 0.134 | -0.093 | NS | -0.250; 0.064 | 0.183 | 0.10 | -0.022; 0.387 |
| ***Mode and frequency of administration*** | | | | | | | | | |
| Single operation | Reference level | | | | | | | | |
| Lumbar puncture 6 times a year | -0.699 | 0.01 | -0.871; -0.527 | -0.735 | 0.01 | -0.955; -0.514 | -0.665 | 0.01 | -0.949; -0.382 |
| Lumbar puncture 12 times a year | -1.476 | 0.01 | -1.661; -1.290 | -1.383 | 0.01 | -1.621; -1.146 | -1.687 | 0.01 | -1.998; -1.376 |
| ***Chance of beneficial effect*** | | | | | | | | | |
|  | 0.075 | 0.01 | 0.067; 0.084 | 0.070 | 0.01 | 0.059; 0.081 | 0.086 | 0.01 | 0.070; 0.101 |
| ***Risks*** | | | | | | | | | |
| 1% risk | Reference level | | | | | | | | |
| 10% risk | -0.061 | NS | -0.233; 0.111 | 0.050 | NS | -0.169; 0.268 | -0.238 | NS | -0.527; 0.051 |
| Unknown long-term risk | -0.097 | NS | -0.260; 0.067 | 0.118 | NS | -0.092; 0.328 | -0.451 | 0.01 | -0.721; -0.182 |
| ***Follow-up*** | | | | | | | | | |
| Nearest local hospital | Reference level | | | | | | | | |
| Nearest university hospital | -0.024 | NS | -0.245; 0.196 | 0.009 | NS | -0.270; 0.287 | -0.076 | NS | -0.447; 0.295 |
| Nurse expert center | 0.145 | NS | -0.063; 0.353 | 0.187 | NS | -0.077; 0.452 | -0.052 | NS | -0.295; 0.398 |
| Neurologist expert center | 0.059 | NS | -0.164; 0.282 | 0.122 | NS | -0.168; 0.411 | -0.045 | NS | -0.403; 0.312 |
|  | | | | | | | | | |
| Log Likelihood | -854.612 |  |  | -518.441 |  |  | -324.393 |  |  |
| Chi squared | 686.272 | 0.000 |  | 358.752 | 0.000 |  | 347.545 | 0.000 |  |
| Adjusted pseudo R^2^ | 0.36 |  |  |  |  |  |  |  |  |
| AIC/N | 1.000 |  |  | 1.047 |  |  | 0.926 |  |  |
| Number of responders* | 216 |  |  | 126 |  |  | 90 |  |  |
| Number of observations | 1728 |  |  | 1008 |  |  | 720 |  |  |

* The 12 respondents who filled in ‘’other underlying disease’’ were added to the SCA subgroup for statistical analysis, except for the premanifest HD patient.

NS = not significant
